# S6. EARLY LIFE ADVERSITIES AND SOCIAL COGNITIVE DYSFUNCTION IN SCHIZOPHRENIA AND OTHER MAJOR PSYCHIATRIC DISORDERS: A SYSTEMATIC REVIEW

**DOI:** 10.1093/schbul/sby018.793

**Published:** 2018-04-01

**Authors:** Karolina Rokita, Maria M Dauvermann, Gary Donohoe

**Affiliations:** 1 Cognitive Genetics and Cognitive Therapy Group, National University of Ireland, Galway; 2 National University of Ireland, Galway

## Abstract

**Background:**

Early life adversity has been identified as a potentially causal factor in the development of mental disorders. Little is known, however, about the association between various types of early life adversities and social cognitive function in adults with major psychiatric disorders, such as schizophrenia, borderline personality disorder, bipolar disorder and major depressive disorder. We conducted a systematic review aimed at elucidating possible underlying cognitive mechanisms that may form the pathway between early life adversities and social cognitive dysfunction.

**Methods:**

Relevant studies were identified via electronic and manual searches of the literature, and included peer reviewed English language articles published up to May 2017. Quality of individual articles was assessed using the quality evaluation scale.

**Results:**

A total of 15 studies were included in the systematic review with the quality assessment scores ranging from 2 to 5 (out of 6). The majority of the studies demonstrated that various types of early life adversities, specifically physical neglect, emotional and sexual abuse and insecure attachment, are significantly associated with social cognitive function.

**Discussion:**

Presented in the context of an attachment model, we conclude that childhood adversity results in poor internal working models, selective attention towards emotional stimuli and greater difficulties with emotional self-regulation. The importance of these findings for development of interventions which diminish the adverse effects of childhood maltreatment on social cognition is discussed.

